# Iodoform Gauze in Ruptured Uterus

**Published:** 1898-07-23

**Authors:** 


### IODOFORM GAUZE IN RUPTURED UTERUS.

What to do in a case of rupture of the uterus is always a matter of anxiety. Supposing the rupture to occur before delivery, and the child to escape into the abdomen, the only chance for the patient clearly lies in immediate abdominal section. But there are cases in which the rupture takes place during delivery, and may, perhaps, only be discovered afterwards in consequence of the hæmorrhage and increasing shock pointing to something having gone wrong. The rupture may not extend down to the lower segment of the cervix, and to discover the rent it may be necessary to introduce the finger within the patulous os. In dealing with such cases, Mayo Robson[1] advises packing with iodoform gauze. The case he gives in illustration was one of hæmorrhage following an abortion at the third month, in which some difficulty had been experienced in removing the ovum, and the cervix had been dilated with Barnes's bags. The vagina was found intact, and also the os and cervix, but on pulling down the cervix by means of the vulsellum forceps, the finger readily passed through a considerable rent in the uterus direct into the peritoneal cavity, and, on pressure over the hypogastric region, blood was forced through the rent. Mr. Robson divided the portion of cervix between the laceration and the vagina, so as to get thoroughly at the rent. This, however, extended too far up for it to be possible to apply sutures so as to effectually occlude it, so, after curetting and washing out the fundus of the uterus, and pressing as much blood as possible out of the abdomen, he packed the laceration very thoroughly with long strips of iodoform gauze, several yards being used, the packing filling the pouch of Douglas, the whole of the laceration, and the upper part of the vagina. The packing was changed on the third day, and after that every second day. There was no further bleeding, no peritonitis, and the patient got quite well.

[1] Practitioner, July, 1898.

